# Physiological Performance, Antioxidant and Immune Status, Columnaris Resistance, and Growth of Nile Tilapia That Received *Alchemilla vulgaris*-Supplemented Diets

**DOI:** 10.3390/antiox11081494

**Published:** 2022-07-29

**Authors:** Abdallah Tageldein Mansour, Heba H. Mahboub, Gehad E. Elshopakey, Enas K. Aziz, Adnan H. M. Alhajji, Gamal Rayan, Hesham S. Ghazzawy, Walaa El-Houseiny

**Affiliations:** 1 Aquaculture and Animal Production Department, Agricultural & Food Sciences College, King Faisal University, P.O. Box 400, Al-Ahsa 31982, Saudi Arabia; aalhajji@kfu.edu.sa (A.H.M.A.); gahmed@kfu.edu.sa (G.R.); 2 Fish and Animal Production Department, Faculty of Agriculture (Saba Basha), Alexandria University, Alexandria 21531, Alexandria, Egypt; 3 Department of Fish Diseases and Management, Faculty of Veterinary Medicine, Zagazig University, Zagazig 44511, Sharkia, Egypt; hhhmb@yahoo.com; 4 Department of Clinical Pathology, Faculty of Veterinary Medicine, Mansoura University, Mansoura 35516, Dakahlia, Egypt; gehadelshobaky@yahoo.com; 5 Department of Husbandry and Animal Wealth Development, Faculty of Veterinary Medicine, University of Sadat City, El Sadat City 23897, Monufia, Egypt; enas.aziz@vet.usc.edu.eg; 6 Date Palm Research Center of Excellence, King Faisal University, Hofuf 31982, Saudi Arabia; hghazzawy@kfu.edu.sa

**Keywords:** *Alchemilla vulgaris*, feed additive, Nile tilapia, immune and antioxidant status, bacterial challenge, growth performance

## Abstract

The current perspective is a pioneering trial to assess the efficacy of the dietary supplementation of *Alchemilla vulgaris* powder (AVP) in the diet of Nile tilapia (*Oreochromis niloticus*) on growth performance, blood picture, hepatic and renal biomarkers, immune status, and serum and tissue antioxidant capacity and to investigate the resistance against *Flavobacterium columnare* challenge. Fish (*n* = 360) were distributed into six groups (three replicates each) and received increasing AVP supplementation levels (0, 2, 4, 6, 8, and 10 g kg^−1^) for 60 days. Furthermore, fish were exposed to the bacterial challenge of a virulent *F. columnare* strain and maintained under observation for 12 days. During the observation period, clinical signs and the cumulative mortality percentage were recorded. The results demonstrated that the growth performance, feed conversion ratio, and hematological profile were noticeably enhanced in the AVP-supplemented groups compared to the control. The most promising results of weight gain and feed conversion ratio were recorded in the groups with 6, 8, and 10 g AVP kg^−1^ diets in a linear regression trend. The levels of hepatorenal function indicators were maintained in a healthy range in the different dietary AVP-supplemented groups. In a dose-dependent manner, fish fed AVP dietary supplements displayed significant augmented serum levels of innate immune indicators (lysozyme, nitric oxide, and complement 3) and antioxidant biomarkers (Catalase (CAT), superoxide dismutase (SOD), total antioxidant (TAC), and reduced glutathione (GSH) with a marked decrease in myeloperoxidase (MPO) and malondialdehyde (MDA) levels). Likewise, hepatic CAT and SOD activities were significantly improved, and the opposite trend was recorded with hepatic MDA. The highest AVP-supplemented dose (10 g/kg) recorded the highest immune-antioxidant status. Based on the study findings, we highlight the efficacy of AVP as a nutraceutical dietary supplementation for aquaculture to enhance growth, physiological performance, and immune-antioxidant status and as a natural economic antibacterial agent in *O. niloticus* for sustaining aquaculture. It could be concluded that the dietary supplementation of 10 g AVP/kg enhanced *O. niloticus* growth, physiological performance, immune-antioxidant status, and resistance against *F. columnare.*

## 1. Introduction

Fish play a significant role in improving human nutrition by providing easily digested animal protein and are considered the main source of long-chain polyunsaturated n-3 fatty acids, which are involved in various metabolic functions [1,2]. Nile tilapia (*Oreochromis niloticus*) is the most common cultured species in Egypt and the third most common in the world because of its rapid growth rate, high feed efficiency, natural spawning, and tasty edible parts [3].

The World Health Organization has declared the spreading of multi-resistant bacterial strains. To avoid the resistance of the pathogen to antibiotics [4], plants are a non-antibiotic, safe, and cheap source that exhibits potent antibacterial activity in aquaculture [5,6,7,8]. Another significant reason for utilizing plant extracts is their natural multi-constituents that are well-documented and verified for promising fish growth and immune-antioxidant traits [9,10,11]. In addition, they protect farmed fish exposed to biotic and abiotic stressors [12], which negatively affect the performance of fish [13].

Among plant extracts, *Alchemilla vulgaris*, commonly known as lion’s foot, bear’s foot, or lady’s mantle, is extensively utilized in public medicine and belongs to the rose family (Rosaceae) [14]. It has been characterized by strong antioxidant activity due to its phenolic constituents [15] and because of the presence of large quantities of tannins (mainly agrimoniin), flavonoids (kaempferol, quercetin, and their glycosides), and phenolcarboxylic acids (caffeic acid, gallic, and ellagic) documented in *A. vulgaris* [16]. *A. vulgaris* could be considered one of the main sources of vegetable tannin, which could be up to 1900 kg/ha [17].

Recently, it has been utilized as a diuretic and in the therapy of hepatic inflammation, asthma, and diabetes as well as renal, gastric, and intestinal disorders [18]. In addition, *A. vulgaris* is used for the treatment of anemia [19]. The potential antibacterial activity of *A. vulgaris* was verified in vitro [20,21] against *Salmonella typhimurium, Enterococcus faecalis, Micrococcus lysodeikticus,* and *Bacillus mycoides* [22].

Columnaris disease affects all stages of life in farmed Nile tilapia fish [23]. It has global economic implications and has resulted in catastrophic mortality up to 100% [24]. Infections are spread either directly or indirectly through contact with unhealthy fish or through the aquatic environment and coexistence with diseased fish that shed bacteria into the water [25]. *F. columnare* has a variety of virulence characteristics, including a strong ability to adhere to fish tissues, the generation of extracellular proteases, and the existence of numerous virulence genes, all of which contribute to its pathogenicity in fish [26]. Consequently, researchers are looking forward to the establishment of efficient control strategies for columnaris disease by creating nutritional techniques that would provide dual benefits as prophylactics for bacterial diseases while improving the health and performance of cultured fish species [27,28].

To the best of our knowledge, there are no reports investigating the efficacy of *Alchemilla vulgaris* powder (AVP) as a dietary supplementation in aquaculture. Therefore, the current study aimed to assess the administration of different concentrations of AVP in *O. niloticus* diets. We wanted to address its impacts on growth performance; hematological, immunological, and antioxidant biomarkers; hepatorenal function; and fish resistance to *F. columnare* infection.

## 2. Materials and Methods

### 2.1. Tested Compounds and Chemicals

Whole *Alchemilla vulgaris* plants (aerial parts and roots) were collected during the full flowering period in midsummer 2020 at Zagazig city, Sharkia province, Egypt. Taxonomic identification was provided by the Botany Department of the Faculty of Agriculture of the University of Zagazig. The collected plant material was air-dried, ground using a laboratory blender to obtain a fine powder, packaged, and kept at 4 °C until it was used. The phytochemical composition of *A. vulgaris* was previously reported according to ref. [16,22], including tannins, flavonoids, and phenolcarboxylic acids. All other analytical-grade chemicals were purchased from Sigma-Aldrich, St. Louis, MO, USA.

### 2.2. Experimental Fish

Apparently healthy Nile tilapia, *Oreochromis niloticus*, fingerlings (average body weight: 22.66 ± 0.75 g/fish) were acquired from a commercial fish farm in Egypt’s Kafr El-Sheik governorate. The fish were diagnosed to be free from ecto-parasitic infestation and flavobacterium infection. Fish were stocked and acclimated for two weeks in 75 L glass aquaria (80 × 40 × 30 cm) with dechlorinated tap water and continuous aeration delivered by an air stone from a central air compressor before the trials, during which they were nourished on a basal diet. After the acclimation period, Nile tilapia fingerlings were distributed into 18 glass aquaria (three replicates/treatment with initial stocking densities of 20 fish/aquarium). Three times a week, around 30% of the water was drained to remove excreta and replaced with dechlorinated tap water. Fish were subjected to a photoperiod of 12 h of light and 12 h of darkness. The physicochemical parameters of the water used in the aquaria were assessed. The temperature was kept at 25 ± 0.36 °C, and the pH and dissolved oxygen levels were kept at 7.25 ± 0.4 and 7.2 ± 0.25 mg/L. The averages of nitrite, ammonia, and nitrate were estimated to be 0.12 ± 0.03, 0.11 ± 0.05, and 2.40 ± 0.04 mg/L.

The study was applied at the Faculty of Veterinary Medicine, Zagazig University, Egypt, in the Fish Diseases and Management Department. Our institution’s Animal Use in Research Committee (IACUC) authorized the experiment. The experiments were completed in compliance with the National Institutes of Health’s (NIH) Ethical Guidelines for the Use and Care of Laboratory Animals in Scientific Investigations.

### 2.3. Diet Formulation

Six experimental diets were used. The control group (AVP0) was fed a basal diet without additives. The second, third, fourth, fifth, and sixth groups were supplemented with AVP diets with various concentrations [2 (AVP2), 4 (AVP4), 6 (AVP6), 8 (AVP8), and 10 g kg^−1^ (AVPP10)].

After thoroughly mixing the ingredients of the experimental diet, they were mechanically pelleted, air-dried at room temperature for 24 h, and then stored at 4 °C until use. The basal diet was designed to meet the optimal nutrient requirements of fish, as recommended by the Nutrient Requirements of Fish [29]. Each experimental diet was tested for moisture in a drying oven, crude protein in a macro-Kjeldahl, crude fat with an ether extraction method, total ash in a muffle furnace, and crude fiber following AOAC [30] guidelines. The ingredients and proximate chemical analysis of the experimental diets are shown in Table 1. Fish were fed at the rate of 5% of their live body weight three times daily (9:00, 12:00, and 16:00). The trial lasted 60 days, and the total fish weight in each aquarium was measured every two weeks to monitor fish growth.

### 2.4. Growth Performance Assessment

At the outset of the experiment and every two weeks thereafter, the fish in each replicate were counted and weighed to determine growth performance. The final body weight (FBW), weight gain (WG), weight gain percentage, specific growth rate (SGR), feed intake (FI), feed conversion ratio (FCR), and condition factor (K) were calculated as follows:

FBW = Total weight of fish/the number of fish in each replicate(1)

WG = Total body weight − initial body weight weight gain percentage = [(Final average body weight-Initial average body weight)/Initial average body weight] × 100(2)

SGR = [(log final body weight-log initial body weight)/time (days)] × 100(3)

FI = feed consumed/Number of survival fish.(4)

FCR = feed fed (g)/weight gain (g).(5)

Condition factor (K) = (Weight/length^3^) × 100. where weight = total weight of fish (g) and L = length of fish (cm), which is measured from the tip of the snout to the end of the middle caudal fin.(6)

### 2.5. Blood and Tissue Sampling

At the end of the 60 day feeding period, blood was collected from the caudal veins of 10 anesthetized fish (50 mg/L clove oil) and divided into two halves. For the first part, EDTA tubes were used to acquire a complete blood cell count. The second sample was taken without anticoagulant to develop the clot, and the tubes were kept at 4 °C overnight and centrifuged at 150 g for 5 min and then 350 g for 15 min. The immunological function, serum protein electrophoretic pattern, oxidant/antioxidant status, and liver and kidney functions were all assessed in the serum. The sampled fish were killed by neck incision and dissected to retrieve the liver. In ten volumes of phosphate-buffered saline, the tissues were homogenized (pH 7.4). The homogenates were then centrifuged at 664 g at 4 °C for 30 min, and the supernatants were stored at −80 °C until further investigation. The liver homogenates were utilized to perform oxidant/antioxidant status tests.

### 2.6. Estimation of Hematological Parameters

The red blood cell counts (RBCs), packed cell volume (PCV), hemoglobin concentration (Hb), mean corpuscular volume (MCV), mean corpuscular hemoglobin (MCH), and mean corpuscular hemoglobin concentration (MCHC) were determined using a Hema Screen 18 automatic hematology analyzer (Hospitex Diagnostics, Sesto Fiorentino, Italy), according to the method described by Feldman, et al. [31]. The total leukocytes (WBCs) and differential leukocyte counts (neutrophils, lymphocytes, and monocytes) were all counted manually using Dacie and Lewis’s approach [32].

### 2.7. Biochemical Analysis

#### 2.7.1. Hepatorenal Function Indicators

Aspartate aminotransferase (AST), alkaline phosphatase (ALP), alanine aminotransferase (ALT), creatinine, and urea were assessed in serum with Spinreact kits (Esteve De Bas, Girona, Spain) in accordance with the protocols proven by Burtis and Ashwood [33], Wenger, et al. [34], Murray and Kaplan [35], Neely and Phillipson [36], and Martinen [37], respectively. The electrophoretic pattern of serum proteins, including total proteins, albumin, and total globulins, was evaluated as described by [38].

#### 2.7.2. Immune System Response

Selective immunological parameters, including complement 3, were evaluated using a Fish Complement 3, C3 ELISA Kit (Code: CSB-E09727s) following the manufacturer’s instructions. Meanwhile, serum lysozyme activity was determined by a turbidimetric assay using spectrophotometry [39]. The method of Montgomery and Dymock [40] was used to determine the concentration of nitric oxide (NO).

#### 2.7.3. Oxidant/Antioxidant Stress Biomarkers

Indicators of stress were measured using colorimetric commercial kits purchased from Bio-diagnostic Co. (Cairo, Egypt). CAT was evaluated according to the method of Aebi [41]. The method is based on the decomposition of H_2_O_2_ by catalase. A known concentration of H_2_O_2_ was present during the incubation of the sample containing catalase. Sodium azide was used to quench the reaction after exactly one minute of incubation. The oxidative coupling reaction of 4-aminophenazone and 3,5-dichloro-2-hydroxybenzenesulfonic acid in the presence of H_2_O_2_, which was catalyzed by horseradish peroxidase, was then used to quantify the amount of H_2_O_2_ still present in the reaction mixture. The resultant quinoneimine dye was measured at 510 nm. The CAT activity was expressed as μmol/mg protein.

The activity of superoxide dismutase (SOD) was measured using the method of Nishikimi, et al. [42]. The technique is based on the SOD enzyme’s capacity to prevent the nitroblue tetrazolium dye from being reduced by phenazine methosulphate. A pH 8.5 buffer, 0.1 mL of nitroblue tetrazolium (NBT), and 0.1 mL of NADH were combined with 0.05 mL of sample. The reaction was started by adding 0.01 mL of phenazine methosulphate (PMs), and the absorbance was then raised and measured for 5 min at 560 nm. SOD activity was expressed as units/mg protein. In order to estimate the level of reduced glutathione (GSH), 0.2 mL of sample homogenate and 3 mL of the precipitating agent, sulfosalicylic acid, were combined with 1.8 mL of distilled water. This mixture underwent 3000× *g* centrifugation for 4 min. The supernatant was then mixed with 4.5 mL of Ellman’s reagent in a volume of 0.5 mL. Then, 0.5 mL of the diluted precipitating agent, 4 mL of phosphate buffer, and 0.5 mL of Ellman’s reagent were combined to create a blank. Within 30 min of color development, the reaction mixture’s absorbance at 412 nm was measured against a control sample. The GSH standard curve was used to extrapolate the GSH concentration [43]. The serum total antioxidant capacity (TAC) was estimated with colorimetric commercial kits (Bio diagnostic Co., Cairo, Egypt). The determination of the antioxidative capacity was performed by the reaction of antioxidants in the sample with a defined amount of exogenously provided H_2_O_2_. A portion of the hydrogen peroxide supplied was removed by the antioxidants in the sample. By means of an enzymatic reaction, including the creation of a colored product from 3,5, dichloro-2-hydroxy benzensulphonate, the residual H_2_O_2_ was measured colorimetrically.

Malondialdehyde (MDA) was measured using the method by Uchiyama and Mihara [44]. By employing a spectrophotometer and the thiobarbituric acid method, the MDA assay in serum was examined. Trichloroacetic acid (TCA) in the amount of 200 microliters was added to the sample, which was then centrifuged at 5000× *g* for 10 min. The pellet was then discarded, and 0.4 mL of thiobarbituric acid (TBA) reagent was added. For 10 min, the solution was incubated in a bath of hot water to produce a pink tint. Using a spectrophotometer, materials were scanned at 532 nm after cooling to ambient temperature. The myeloperoxidase (MPO) activity in the serum of fish was determined using the method of Kumari and Sahoo [45]. First, 30 µL of serum was diluted with 370 mL of HBSS devoid of Ca^2+^ or Mg^2+^ in Eppendorf tubes. A mixture of 0.006 percent fresh hydrogen peroxide and 100 µL of 3,3′,5,5′-tetramethylbenzidine dihydrochloride at 0.1 mg/mL each was added. By monitoring the increase in absorbance, the process was followed kinetically. The amount of enzyme needed to produce a 0.001 rise in absorbance per minute in 0.5 mL of the reaction mixture (A 450/min/mL) was used to calculate the reaction velocities.

### 2.8. Pathogenic Bacteria and Immersion Challenge Test

*Flavobacterium columnare* was previously isolated from diseased *O. niloticus* and was previously identified and validated as pathogenic. The isolate was presumptively defined by the biochemical approach of Griffin [46] using both cultural characteristics and conventional biochemical assays. The isolate was withdrawn out of the freezer at −80 °C and streaked on Anacker and Ordal’s medium. Using a sterile cotton swab, the isolate was dislodged from the agar and transferred to *F. columnare* growth medium broth (FCGM) [47]. The inoculated broth was incubated at 28 °C for 24 h with continuous shaking at 100 rpm.

At the end of the feeding trial (60 days), 10 fish from each treatment were randomly selected and were challenged by the immersion method in a bucket (10 L of continuously aerated water) containing the bacterial broth for 2 h with a final concentration of 1.5 × 10^8^ CFU/mL (a sub-lethal concentration). The lethal dose (LD_50_) of *F. columnare* was first estimated to determine the sub-lethal dose suitable for the challenge by bathing fish in a 10 L water bucket of five different concentrations of live bacteria for 24 h. The actual concentration of *flavobacterium inoculum* was determined by the standard plate count method on specific agar. The mortality of infected fish was recorded seven days after the challenge, and the LD_50_ was calculated as 5.7 × 10^8^ CFU/mL. The two-hour exposure time was based on preliminary experiments indicating that 2 h was necessary to produce an acute infection. After the challenge, fish were removed from the challenged suspensions, returned to their respective tanks, and maintained under normal husbandry conditions. The challenged fish were monitored for twelve days to observe the signs of columnaris disease. The daily and accumulative mortality were tracked to preliminarily evaluate the effects of the six diets. The isolation of *F. columnare* from moribund and dead fish to confirm columnaris as a cause of death was performed.

### 2.9. Statistical Analysis

The data were statistically investigated using a one-way analysis of variance (ANOVA) (SPSS version 16.0, SPSS Inc., Chicago, IL, USA). Tukey’s multiple comparisons post hoc test was used to compare the differences between groups, with statistical significance set at *p* < 0.05. The results of the analysis are presented as means ± SE (standard error). In addition, a fit regression model was created between the increasing dietary levels of AVP, and weight gain and FCR were estimated using SPSS version 16, SPSS Inc., Chicago, IL, USA.

## 3. Results

### 3.1. Growth Performance and Feed Utilization

The final weight (FW), weight gain (WG), weight gain % (WG %), and specific growth rate (%) (SGR) were meaningfully (*p* < 0.05) enhanced by supplementing AVP at 6–10 g/kg (Table 2). Feed intake was significantly increased with increasing AVP supplementation levels in the fish diet. Further, the condition factor (K) was markedly increased in the AVP8 and AVP10 groups compared to the other groups.

**Figure 1:**
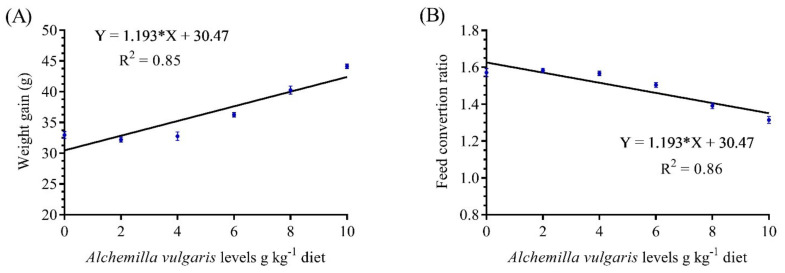
Fit linear regression models of weight gain (**A**) and feed conversion ratio (**B**) in response to increasing dietary supplementation of *Alchemilla vulgaris* (g/kg).

Fish fed AVP-supplemented rations expressed significantly better FCR values. Additionally, the lowest FCR values were achieved by those fish fed 10 g/kg, while the highest FCR values were obtained in AVP2 group. The maximum growth performance was obtained in the group fed 10 g/kg, while the lowest one was obtained in the control group without AVP supplementation (Table 2). The dose–response study showed a linear increasing trend of WG and FCR in response to the increasing dietary supplementation of AVP, with a strong correlation (R^2^ = 0.85) (Figure 1).

### 3.2. Hematological Indices

The effects of AVP supplementation in the diets of *O. niloticus* fish on hematological indices are presented in Table 3. The erythrogram revealed that RBCs, Hb, and PCV were significantly higher in the AVP6, AVP8, and AVP10 groups compared to the other groups. On the other hand, there were no significant differences between the treatments for MCV and MCH. Marked increases in the total WBCs, lymphocytes, neutrophils, eosinophils, and monocyte levels were observed as a result of increasing the concentration of AVP from 6 to 10 g/kg. Furthermore, fish treated with 10 g/kg had higher leukogram indices.

### 3.3. Hepatorenal Function Indicators

The effects of the dietary supplementation of AVP on Nile tilapia serum biochemical parameters are shown in Table 4. The serum total protein of fish was significantly influenced by the dietary treatments. The serum total protein and albumin were increased in a level-dependent manner with the increasing dietary supplementation of AVP. Fish fed 8 and 10 g/kg AVP experienced significant drops in ALT, AST, ALP, urea, and creatinine levels. Fish fed diets of 0-6 g AVP/kg displayed the highest levels of the same parameters.

### 3.4. Innate Immune Response

**Figure 2:**
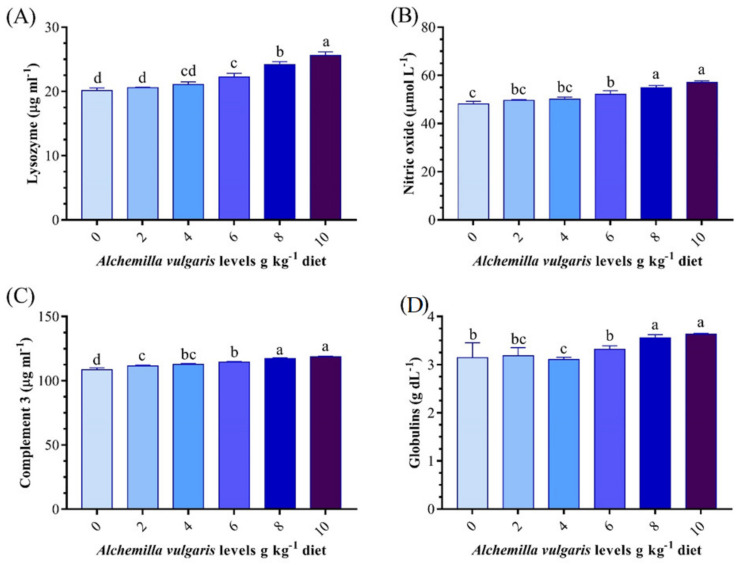
Effect of *Alchemilla vulgaris* dietary supplementation on innate immune parameters of *O. niloticus* for 60 days. (**A**) lysozyme, (**B**) nitric oxide, (**C**) complement 3, (**D**) globulin. Columns bearing different letters are significantly different at *p* < 0.05.

The innate immunity parameters (lysozyme, C3, and NO) were significantly influenced by the experimental treatments (Figure 2). The lysozyme activity, C3, and NO levels were increased significantly with AVP doses over a 4 g/kg diet. The highest immune status was recorded in the group fed the 10 g AVP/kg diet. The globulin level was increased significantly in groups fed 8–10 g AVP/kg^1^ diets compared to the other treatments.

### 3.5. Antioxidant and Oxidative Stress Biomarkers

**Figure 3:**
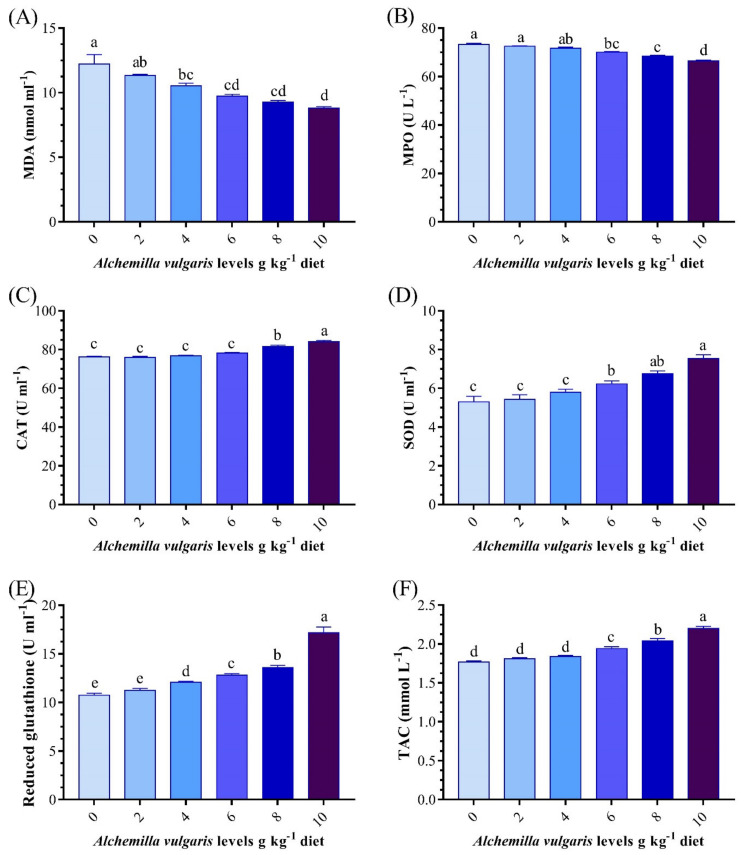
Effect of *Alchemilla vulgaris* dietary supplementation on antioxidant biomarkers in the serum of *O. niloticus*. (**A**) Malondialdehyde, (**B**) myeloperoxidase, (**C**) catalase, (**D**) superoxide dismutase, (**E**) reduced glutathione, (**F**) total antioxidant capacity. Columns bearing different letters are significantly different at *p* < 0.05.

**Figure 4:**
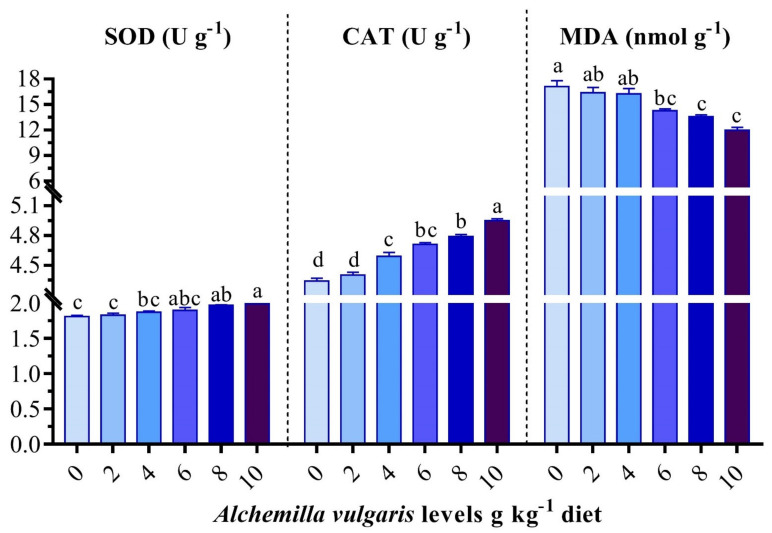
Effect of *Alchemilla vulgaris* dietary supplementation on antioxidant biomarkers in the liver homogenate of *O. niloticus*. SOD: superoxide dismutase, CAT: catalase, MDA: malondialdehyde. Columns bearing different letters are significantly different at *p* < 0.05.

The SOD, CAT, and MPO activities and GSH, TAC, and MDA levels were significantly changed from the controls in the groups receiving the experimental diets (Figure 3). The groups of fish that received dietary AVP at rates of 8 and 10 g/kg showed pronounced increases (*p* < 0.05) in the serum SOD, CAT, GSH, and TAC activities (Figure 3). Nevertheless, the AVP-supplemented diets decreased stress, as conveyed by the gradual drop in MDA and MPO. The best results were obtained in the AVP10 group. Likewise, the hepatic CAT as well as SOD activities in AVP6, AVP8, and AVP10 were significantly higher than those of the other groups. Meanwhile, the opposite trend was observed for the MDA content (Figure 4).

### 3.6. Fish Resistance against Challenge with Flavobacterium columnare

**Figure 5:**
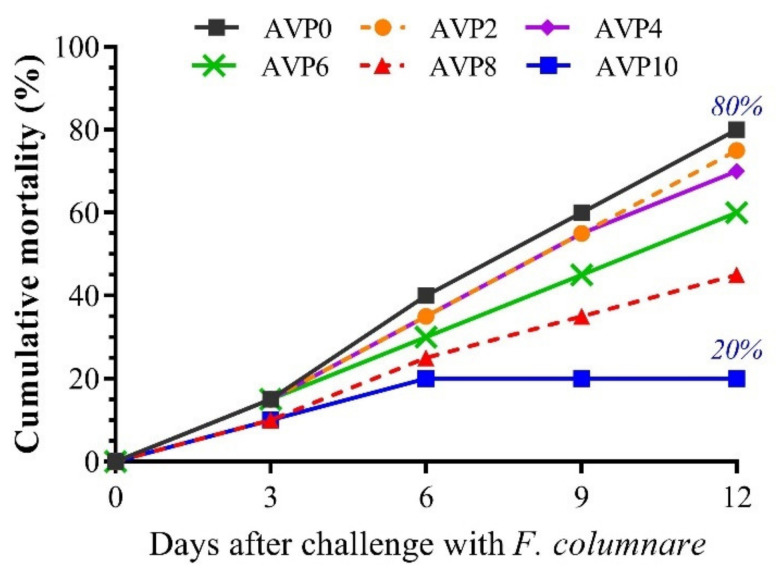
Effect of increasing dietary levels of *Alchemilla vulgaris* on cumulative mortality percent of *Oreochromis niloticus* challenged with pathogenic *Flavobacterium columnare* for 12 days.

To further assess the impact of AVP supplementation on the efficiency of the immune system of *O. niloticus*, fish were challenged with *F. columnare* (Figure 5). The cumulative mortality percent in the AVP0 group reached 80% after 12 days of the challenge. Meanwhile, the dietary supplementation with AVP decreased the cumulative mortalities in a dose-dependent manner. The lowest cumulative mortality (20%) was recorded in the group that was fed a 10 g AVP/kg diet.

**Figure 6:**
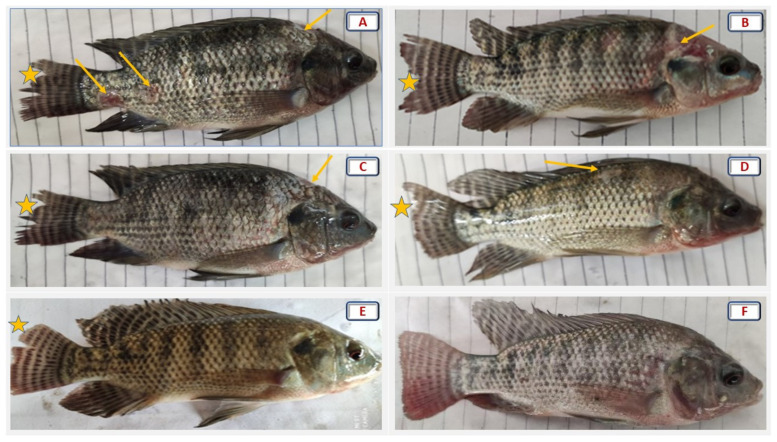
Effect of increasing dietary levels of *Alchemilla vulgaris* on columnaris clinical symptoms of *Oreochromis niloticus* challenged with pathogenic *Flavobacterium columnare* for 12 days. Stars identify fin rots, and arrows identify pale ulcers with hemorrhagic boundaries, especially on the upper head (saddleback lesion) and caudal peduncle. (**A**) 0 g AVP/kg diet, (**B**) 2 g AVP/kg diet, (**C**) 4 g AVP/kg diet, (**D**) 6 g AVP/kg diet, (**E**) 8 g AVP/kg diet, (**F**) 10 g AVP/kg diet.

As shown in Figure 6, different degrees of fin rot, hemorrhage, and pale ulcers with hemorrhagic boundaries, especially on the upper head (saddleback lesion) were evident in the challenged groups fed 0–6 g AVP/kg. Meanwhile, the groups fed 8 and 10 g/kg revealed clear improvements in fish resistance to *F. columnare* infection. Notably, the AVP10 group showed the highest recovery and the lowest cumulative mortality percentage after the bacterial challenge.

## 4. Discussion

In the last decade, great changes have occurred in classic medicine, which have had a great influence on the studies of natural extracts as potential remedies for several animal and human diseases [48]. The *Alchemilla vulgaris* powder, as a promising source of feed supplement, contains high levels of polyphenolic components (395.65 mg/100 g) and flavonoid content (183.10 mg/100 g), including benzoic acid (1084.63 ppm), ellagic acid (614.16 ppm), catechol (580.54 ppm), catechin (566.53 ppm), salicylic acid (479.71 ppm), and protocatechuic acid (444.43 ppm) [16]. Accordingly, the present work was established to evaluate AVP as an available and cheap plant with significant nutritional importance to sustain aquaculture by improving fish performance and health and strengthening the resistance against *F. columnare*. There are no reports elucidating AVP usage in aquaculture. Accordingly, this study highlights the effect of AVP on growth performance, blood picture, immune-antioxidant-associated indices, hepatorenal functions, and *F. columnare* resistance in *O. niloticus* in response to different AVP-incorporated diets.

In aquaculture, medicinal plants and their extracts display a significant role in modulating growth performance [38]. Herein, the results reveal better growth performance and feed utilization in all AVP-supplemented groups, and the best results were recorded at higher doses of supplementation. Accordingly, the dietary supplementation of AVP improved the weight gain and FCR of heat-stressed broilers [49]. In addition, AVP at a dose of 3% improved quail production and the feed conversion ratio and mitigated the negative effects of high temperature [50]. This could be dominated by the potent influence of AVP on digestive activity, which thereby has a positive effect on growth and feed utilization. Our findings are supported by Samah et al. [51], who stated that AVP has a direct impact on digestive enzyme activities, which enhance digestion and consequently reflect a better growth rate. In addition, tannin, as the main active component of AVP, has a positive influence on fish growth performance. In the same line, the dietary *Elephantopus scaber* extract, a rich source of flavonoids and phenols, significantly improved the WG, SGR, and FCR of Nile tilapia at the level of 5 g/kg [52]. The dietary supplementation of condensed tannin (1 g/kg) improved growth performance and decreased the serum glucose concentration of *Lateolabrax japonicus* [53], which could be due to the regulation effect of tannin on the activity and gene expression of hepatic glycolysis and gluconeogenesis enzymes [53].

Meanwhile, the growth performance and intestinal histomorphology of *L. japonicus* fed low doses of tannin (lower than 400 mg/kg diet) were not affected [54], and using extremely high levels of tannin in the grass carp (*Ctenopharyngodon idella*) diet (over 20 g/kg diet) caused severe growth suppression and enteritis morbidity [55]. Therefore, the present findings revealed that dietary AVP up to 10 g/kg diet could provide the optimal tannin level for the growth performance and feed utilization of Nile tilapia.

An assessment of fish blood profile is a substantial tool to reflect the efficacy of plant extracts on fish physiological performance [56,57,58]. The present perspective demonstrated elevated levels of hematological parameters, particularly monocytes, reflecting a strong hematological response in association with the AVP dietary supplementation. Similarly, a recent study reported that monocyte levels were notably augmented by increasing the dose of AVP in broiler diets [49]. Furthermore, the MCV, MCH, and platelet distribution width increased significantly in quails fed 1% AVP compared to the control [59]. Such plant extracts could be used to treat anemic cases and stimulate hematopoiesis organs [57]. The phenols, among the other phytochemicals in AVP, could significantly stimulate the synthesis of blood cells. Accordingly, the leukocytes counts were increased significantly in common carp, *Cyprinus carpio,* fed 1.5, 4.5, or 9 g pistachio, *Pistacia vera*/kg diets, as a rich source of phenols, compared to the control [60].

In addition, the hepatorenal functions, as a center of metabolism and excretion processes in the fish body, could be also considered in evaluating any new feed supplement to reveal its safety and/or efficiency for animals. The present results showed that the administration of AVP modulated the levels of liver function enzymes (AST, ALP, and ALT) and kidney function biomarkers (urea and creatinine) and increased the total protein and albumin levels in the serum of *O. niloticus* compared to the control group. In the same line, the dietary supplementation of 100 ppm ethanol leaves extract or 2% *A. vulgaris* powder ameliorated the effect of CCl_4_ toxicity on liver and kidney functions and reduced the liver histopathological and biochemical changes in the intoxicated rats [16]. In addition, Ozbek et al. [61] found that the extracts of *A. mollis* had a hepatoprotective activity via modulating the level of aminotransferases in mouse serum. The improving effect of AVP on liver and kidney functions could be attributed to its phytochemical contents of phenolics and flavonoids that are well-known as strong antioxidants, which could protect the hepatocytes and glomerulus from oxidative damage [62,63]. Dietary flavonoids significantly decreased serum AST and ALT activities in the intoxicated *C. Idella* [64].

*Alchemilla vulgaris* is characterized by potent antioxidant activity due to its richness in phenolics, especially tannins, which are responsible for its antioxidant activity [59,65,66]. The current investigation implied a strong antioxidant response in fish fed AVP-enriched diets, as evidenced by the marked increase in the antioxidant biomarkers (CAT, SOD, TAC, and GSH). In addition, the MDA level and MPO activity were decreased with increasing AVP dietary supplementation. The abovementioned antioxidant effects of AVP could be attributed to its natural bioactive compounds, mostly phenolic compounds, including tannins, phenolcarboxylic acids, and flavonoids [67]. Whereas, Vlaisavljević et al. [66] quantified twenty-six phenolic compounds in AVP-enriched diets and detected its richness in quercetin, catechin, hexoside, apigenin, luteolin, gallic, and caffeic acids, which are proven to play major roles in protecting against oxidative stress. *A. vulgaris* dried leaves and different leaf extracts have high free radical scavenging activities in terms of DPPH [16,68]. In the same line, broilers fed AVP-supplemented diets experienced low serum MDA and suppressed lipid peroxidation in a dose-dependent manner [49].

Increasing the supplementation levels of flavonoids in the diet of snakehead fish (*Channa argus*) significantly enhanced the gene expression and activities of liver antioxidant enzymes (SOD, CAT, and glutathione peroxidase) and reduced the MDA levels in the liver [62]. MDA and protein carbonyl in the livers, spleens, and gills of chromium-intoxicated grass carp, *Ctenopharyngodon idella,* decreased significantly with dietary flavonoids of Allium mongolicum Regel [64]. In addition, natural phenolic compound (derived from mango peel) supplementation into the zebrafish diet significantly decreased muscle and increased CAT activity. However, glutathione peroxidase and SOD activities did not affect phenolic supplementation [69]. The dietary condensed tannin upregulated the hepatic mRNA levels of nuclear factor erythroid 2-related factor 2, heme oxygenase 1, CAT, and SOD in [53]. Several studies support our outcomes and elucidate that plant phytochemicals exhibit strong scavenging activity and are antioxidant system stimulators [22,70,71,72,73].

The non-specific immunity status in fish in response to a dietary supplement represents the first line of defense against all infections and assists the development of a specific immune response [74,75]. In the current study, the activity of lysozyme, nitric oxide, and complement 3 and the globulin level experienced higher values in AVP-incorporated diets, suggesting an immune-modulating impact of AVP and an increased tolerance of the bacterial infection. The promising influence of AVP on the fish immune system could be attributed to its richness in phyto-components such as flavonoids, phenolics, tannins, and ellagic acids [22,76]. Dietary flavonoids significantly increased lysozyme activity and the C3 level in the kidney and intestine in *C. Idella* [64]. Moreover, dietary phenolic extracts of licorice root increased the respiratory burst and lysozyme activities of Nile tilapia and improved fish bacterial resistance [77].

The challenge trial is a validating tool to investigate the fish’s immune-antioxidant function [69]. Fish that received the dietary intervention of AVP for 8 weeks before being challenged with *F. columnaris* exhibited remarkable improvements in the clinical signs, especially at higher supplemented doses. A lower mortality rate was also reported in AVP-enriched diets. This could be dominated by the effect of AVP on elevating monocytes, which are considered blood phagocytic cells that support the immune system by killing pathogenic bacterium and consequently enhance fish resistance, as reported by [78]. *A. vulgaris* showed dose-dependent antiviral and antibacterial properties [20,21]. Moreover, several feed additives revealed significant improvements in fish against *F. columnaris* infection [79,80].

In addition, the noticeable improvement in skin ulcers could be dominated by the impact of the phenolic constituents of AVP on curing ulcers, implying an anti-inflammatory effect. Our findings were supported by [81] who described that this plant is active against inflammation and is used for wound healing due to its potent and anti-inflammatory and antimicrobial impacts. The application of an ointment containing an *Alchemilla vulgaris* and *Mimosa tenuiflora* herbal mixture promoted the migration of keratinocytes, fibroblasts, and endothelial cells and the proliferation of macrophages and lymphatic vessels and induced rapid wound healing [82]. Based on the outcomes, the current study implied that the administration of AVP in *O. niloticus* diets enhanced growth, the hematological response, hepatorenal function, and immunity-antioxidant status. Moreover, it augmented the tolerance of fish against infection with *F. columnaris*.

## 5. Conclusions

Until now, the current study represents a pioneering trial in aquaculture to investigate the efficacy of increasing levels of dietary *Alchemilla vulgaris* powder (AVP) (0, 2, 4, 6, 8, and 10 g/kg diet) on growth, blood picture, immune-antioxidant response, and hepatorenal function and against bacterial infection with *Flavobacterium columnare* (*F. columnare*) in *O. niloticus*. The results revealed that dietary supplements with AVP promoted fish performance, hematological profile, immunity, antioxidant capacity, and liver and kidney functions in a dose-dependent manner. In addition, the AVP-incorporated diets strengthened the resistance against *F. columnare* by alleviating the clinical columnaris symptoms and reducing the mortality rate. The AVP dietary intervention is highly motivated as an environmentally friendly growth stimulator and immune-antioxidant promoter and for the treatment of anemic cases. It can also be utilized as an alternative antibacterial treatment to avoid the resistance of antibiotics, with less hazardous impacts to the environment. Further studies are needed to assess other AVP antimicrobial activities and to test the efficacy of AVP antimicrobial activities in in vitro conditions.

## Figures and Tables

**Table 1:** Ingredients and proximate chemical analysis of the experimental diets (g/kg).

Ingredients (g/kg)	Dietary *Alchemilla vulgaris* Levels (g/kg Diet)					
	0	2	4	6	8	10
Fishmeal	110	110	110	110	110	110
Corn flour	330	330	330	330	330	330
Soybean meal 44%	290	290	290	290	290	290
Corn gluten meal 60%	120	120	120	120	120	120
Wheat bran	80	78	76	74	72	70
Soybean oil	20	20	20	20	20	20
Fish oil	20	20	20	20	20	20
*Alchemilla vulgaris* powder (AVP)	0	2	4	6	8	10
Vitamin premix ^1^	15	15	15	15	15	15
Mineral premix ^2^	15	15	15	15	15	15
Total	1000	1000	1000	1000	1000	1000
Chemical analysis (g/kg)						
Crude protein (*n* × 6.25)	308.8	309.5	309.7	310.0	310.3	310.6
Crude lipids	74.9	75.1	75.7	76.5	78.4	79.1
Crude fiber	52.3	53.4	53.8	54.1	54.5	54.9
Ash	52.2	53.7	54.5	56.9	57.9	58.4
Nitrogen-free extract ^3^	511.8	508.3	506.3	502.5	498.9	497
Gross energy (kcal/kg) ^4^	4556	4547	4546	4539	4544	4545

^1^ Vitamin premix (per kg of premix): vitamin A, 8,000,000 IU; vitamin E, 7000 mg; vitamin D_3_, 2,000,000 IU; vitamin K_3_, 1500 mg; biotin, 50 mg; folic acid, 700 mg; nicotinic acid, 20,000 mg; pantothenic acid, 7000 mg; vitamin B_1_, 700 mg; vitamin B_2_, 3500 mg; vitamin B_6_, 1000 mg; vitamin B_12_, 7 mg. ^2^ Mineral premix (per kg of premix): zinc sulfate, 4.0 g; iron sulfate, 20 g; manganese sulfate, 5.3 g; copper sulfate, 2.7 g; calcium iodine, 0.34 g; sodium selenite, 70 mg; cobalt sulfate, 70 mg; and CaHPO_4_·2H_2_O up to 1 kg. ^3^ Calculated by difference (100 − protein% + lipids% + ash% + crude fiber %). ^4^ Gross energy (GE) was calculated as 5.65, 9.45, and 4.11 kcal/g for protein, lipid, and NFE, respectively (NRC, 1993).

**Table 2:** Effect of *Alchemilla vulgaris* dietary supplementation on growth performance and feed utilization of *O. niloticus*.

Items	Dietary *Alchemilla vulgaris* Levels (g/kg Diet)					
	0	2	4	6	8	10
Final body weight (g)	55.63 ^d^ ± 0.46	55.60 ^d^ ± 0.56	55.50 ^d^ ± 0.55	59.16 ^c^ ± 0.44	63.70 ^b^ ± 0.70	67.0 ^a^ ± 0.57
Weight gain (g)	32.96 ^d^ ± 0.31	32.20 ^d^ ± 0.23	32.76 ^d^ ± 40.0	36.26 ^c^ ± 0.20	40.26 ^b^ ± 0.37	44.13 ^a^ ± 0.20
Daily weight gain (g)	0.54 ^d^ ± 0.005	0.53 ^d^ ± 0.003	0.54 ^d^ ± 0.006	0.60 ^c^ ± 0.003	0.67 ^b^ ± 0.006	0.73 ^a^ ± 0.002
Specific growth rate (%)	0.65 ^c^ ± 0.018	0.62 ^c^ ± 0.007	0.64 ^c^ ± 0.017	0.68 ^bc^ ± 0.014	0.72 ^ab^ ± 0.003	0.77 ^a^ ± 0.015
Feed intake (g)	52.00 ^c^ ± 0.57	51.00 ^c^ ± 0.57	51.33 ^c^ ± 0.44	54.50 ^b^ ± 0.28	56.00 ^ab^ ± 0.28	58.00 ^a^ ± 0.57
Feed conversion ratio	1.57 ^a^ ± 0.012	1.58 ^a^ ± 0.006	1.56 ^a^ ± 0.007	1.50 ^b^ ± 0.007	1.39 ^c^ ± 0.008	1.31 ^d^ ± 0.010
Condition factor (K)	2.02 ^c^ ± 0.008	2.04 ^c^ ± 0.017	2.05 ^c^ ± 0.020	2.065 ^c^ ± 0.010	2.22 ^b^ ± 0.004	2.38 ^a^ ± 0.026

Values are presented as means ± SE, and *n* = 3. The means within the same row carrying different superscripts letters are significant at *p* < 0.05.

**Table 3:** Effect of *Alchemilla vulgaris* dietary supplementation on hematological indices of *O. niloticus*.

Items	Dietary *Alchemilla vulgaris* Levels (g/kg Diet)					
	0	2	4	6	8	10
RBCs (10^6^/mm^3^)	3.78 ^d^ ± 0.008	3.81 ^cd^ ± 0.008	3.84 ^c^ ± 0.008	3.89 ^b^ ± 0.008	3.93 ^ab^ ± 0.005	3.96 ^a^ ± 0.017
Hb (gm/dL)	11.20 ^d^ ± 0.05	11.30 ^cd^ ± 0.02	11.34 ^cd^ ± 0.01	11.44 ^bc^ ± 0.02	11.52 ^b^ ± 0.01	11.80 ^a^ ± 0.04
PCV (%)	33.60 ^d^ ± 0.17	33.92 ^cd^ ± 0.08	34.03 ^cd^ ± 0.04	34.33 ^bc^ ± 0.07	34.57 ^b^ ± 0.04	35.42 ^a^ ± 0.14
MCV(fl)	88.73 ^ab^ ± 0.66	88.87 ^ab^ ± 0.13	88.46 ^ab^ ± 0.10	88.10 ^b^ ± 0.05	87.96 ^b^ ± 0.02	89.44 ^a^ ± 0.06
MCH (%)	29.57 ^ab^ ± 0.22	29.62 ^ab^ ± 0.04	29.48 ^ab^ ± 0.03	29.36 ^b^ ± 0.01	29.32 ^b^ ± 0.007	29.81 ^a^ ± 0.02
WBCs (10^3^/mm^3^)	5.25 ^d^ ± 0.02	5.28 ^d^ ± 0.01	5.32 ^cd^ ± 0.01	5.37 ^bc^ ± 0.008	5.42 ^b^ ± 0.01	5.51 ^a^ ± 0.02
Lymphocytes (10^3^/mm^3^)	2.90 ^c^ ± 0.005	2.90 ^c^ ± 0.008	2.90 ^c^ ± 0.005	2.92 ^bc^ ± 0.005	2.94 ^b^ ± 0.008	2.99 ^a^ ± 0.011
Neutrophils (10^3^/mm^3^)	1.38 ^c^ ± 0.006	1.40 ^bc^ ± 0.005	1.41 ^bc^ ± 0.008	1.43 ^ab^ ± 0.012	1.43 ^ab^ ± 0.005	1.46 ^a^ ± 0.008
Eosinophils (10^3^/mm^3^)	0.33 ^b^ ± 0.005	0.33 ^b^ ± 0.005	0.35 ^ab^ ± 0.008	0.38 ^a^ ± 0.005	0.38 ^a^ ± 0.005	0.37 ^a^ ± 0.005
Monocytes (10^3^/mm^3^)	0.64 ^b^ ± 0.005	0.65 ^b^ ± 0.005	0.65 ^b^ ± 0.011	0.64 ^b^ ± 0.005	0.67 ^ab^ ± 0.005	0.69 ^a^ ± 0.005

Values are presented as means ± SE, and *n* = 10. The means within the same row carrying different superscripts letters are significant at *p* < 0.05.

**Table 4:** Effect of *Alchemilla vulgaris* dietary supplementation on serum biochemical parameters of *O. niloticus*.

Items	Dietary *Alchemilla vulgaris* Levels (g/kg Diet)					
	0	2	4	6	8	10
Total proteins (g/dL)	5.46 ^d^ ± 0.10	6.10 ^c^ ± 0.069	6.13 ^bc^ ± 0.04	6.44 ^b^ ± 0.086	6.81 ^a^ ± 0.060	6.94 ^a^ ± 0.026
Albumin (g/dL)	2.31 ^c^ ± 0.101	2.92 ^b^ ± 0.136	3.03 ^ab^ ± 0.02	3.12 ^ab^ ± 0.06	3.26 ^ab^ ± 0.02	3.31 ^a^ ± 0.014
ALT (U/L)	13.24 ^a^ ± 0.43	13.26 ^a^ ± 0.48	12.45 ^ab^ ± 0.29	11.96 ^ab^ ± 0.12	11.73 ^b^ ± 0.06	11.58 ^b^ ± 0.04
AST (U/L)	27.80 ^a^ ± 0.32	27.70 ^a^ ± 0.32	27.02 ^ab^ ± 0.07	26.85 ^ab^ ± 0.13	26.71 ^b^ ± 0.12	26.15 ^b^ ± 0.09
ALP (IU/L)	24.30 ^a^ ± 0.15	24.21 ^a^ ± 0.06	24.08 ^a^ ± 0.06	23.90 ^ab^ ± 0.09	23.60 ^b^ ± 0.08	23.09 ^c^ ± 0.06
Urea (mg/dL)	2.76 ^a^ ± 0.037	2.76 ^a^ ± 0.023	2.68 ^a^ ± 0.02	2.59 ^a^ ± 0.03	2.18 ^b^ ± 0.044	2.14 ^b^ ± 0.049
Creatinine (mg/dL)	0.436 ^a^ ± 0.01	0.44 ^a^ ± 0.005	0.41 ^ab^ ± 0.01	0.356 ^bc^ ± 0.01	0.323 ^c^ ± 0.01	0.246 ^d^ ± 0.01

Values are presented as means ± SE, and *n* = 10. The means within the same row carrying different superscripts letters are significant at *p* < 0.05.

## Data Availability

The data that support the findings of this study are contained within the article.
